# Expression of PD‐L1 on regulatory B cells in patients with acute myeloid leukaemia and its effect on prognosis

**DOI:** 10.1111/jcmm.17390

**Published:** 2022-05-24

**Authors:** Yingqing Shi, Zhuogang Liu, Hongtao Wang

**Affiliations:** ^1^ 85024 Department of Hematology Shengjing Hospital of China Medical University Shenyang China

**Keywords:** acute myeloid leukaemia (AML), programmed death‐ligand 1 (PD‐L1), regulatory B cells (Breg)

## Abstract

Programmed death‐ligand 1 (PD‐L1) is involved in immunosuppression in variety of tumours. Regulatory B cells (Bregs) are critical immune regulatory cells, and it has been demonstrated that the number of regulatory B cells in patients with acute myeloid leukaemia (AML) is much higher than that in healthy donors (HDs), which is linked to a poor prognosis. This study aimed to determine whether increased expression of PD‐L1, including in Bregs, is associated with a worse prognosis in individuals with AML. The proportion of Bregs, PD‐L1 expression in Bregs and PD‐1 expression in T cells were determined using flow cytometry using patient samples from 21 newly diagnosed AML patients at different stages of treatment and 25 HDs. We confirmed PD‐L1 expression in Bregs, and PD‐1 expression in CD3^+^CD4^+^T cells in bone marrow and peripheral blood samples from AML patients was higher than that in samples from HDs. The complete remission (CR) and progression‐free survival (PFS) of Bregs with high PD‐L1 expression were significantly decreased following induction chemotherapy. PD‐L1 expression is indeed increased in Bregs from individuals with AML, and high PD‐L1 expression is related to a poor prognosis.

## 1 INTRODUCTION

Acute myeloid leukaemia (AML) is the most frequent kind of leukaemia in adults, and its pathophysiology is believed to be linked to immunosuppression, particularly affecting T lymphocytes. There is evidence that acute myeloid leukaemia patients have some degree of T‐cell malfunction, including a loss in their capacity to multiply or produce cytokines, but the number of studies on this topic is limited.^1^, ^2^, ^3^, ^4^, ^5^ T‐cell dysfunction in the tumour microenvironment is a critical method by which tumours evade immune surveillance, allowing cancer cells to evade immune cell assault.

Regulatory B cells (Bregs) are a recently identified subset of critical immune regulatory cells. Although Bregs are few in number, they are critical for immunological balance by increasing the number and function of Treg cells and blocking T cells, mononuclear macrophages and pathogenic B cells from secreting inflammatory cytokines.^6^, ^7^ Blair et al. proved that human CD19^+^ CD24^+^CD38^+^B cells have the ability to negatively regulate T cells.^8^ Our team previously discovered that the expression of CD19^+^CD24^+^CD38^+^ B cells is dramatically enhanced in AML and is associated with a poor prognosis.^9^

Programmed death‐ligand 1 (PD‐L1) binds to programmed death 1 (PD‐1) expressed on T cells, B cells, dendritic cells and natural killer T cells, thereby inhibiting anticancer immunity.^10^ It has been reported that PD‐L1 is highly expressed in Bregs in solid tumours, such as breast cancer and melanoma, and Bregs can immunosuppress T cells through the PD‐L1 pathway, affecting their function.^11^, ^12^, ^13^ Some studies have further confirmed that PD‐L1 can mediate the immunosuppressive effect of Bregs on T cells, which is an IL‐10‐independent pathway.^14^ Therefore, we hypothesized that the immune escape mechanism of PD‐1/PD‐L1 is functional in patients with AML.

At one point, the concept of PD‐L1 protein expression in AML patients was contentious.^15^, ^16^ Therefore, the purpose of this study was to explore the expression of PD‐L1 in patients with AML and to lay a foundation for follow‐up experiments.

## 2 MATERIALS AND METHODS

### 2.1 Enrolled patients

A total of 21 patients with acute myeloid leukaemia diagnosed for the first time at the Department of Haematology of Shengjing Hospital affiliated with China Medical University from May 2021 to September 2021 underwent standard chemotherapy (continuous infusion cytarabine for 7 days and anthracycline for 3 days). Acute myeloid leukaemia diagnosis and classification were made according to the 2016 World Health Organization classification of acute myeloid leukaemia. Bone marrow (BM) was collected from 18 patients. Peripheral blood (PB) was collected from 21 patients at 7 days after chemotherapy, and peripheral blood was collected from 19 patients at 14 days after chemotherapy. In addition, we recruited 15 healthy donors for the collection of bone marrow and 10 healthy donors for the collection of peripheral blood. None of the donors had any immune system abnormalities. This study was approved by the Ethics Committee of Shengjing Hospital affiliated with China Medical University and followed the guidelines outlined in the Declaration of Helsinki (NO:2020PS278K). All participants provided written informed consent prior to registration.

### 2.2 Flow cytometry

Samples (100 µl) collected from bone marrow or peripheral blood were put into two tubes, and the following antibodies were added: FITC‐anti‐CD3, APC‐anti‐CD4, PerCP‐anti‐CD8 and FITC‐anti‐CD24. APC‐anti‐CD38, PerCP‐anti‐CD19, PE‐anti‐PD‐L1 and PE‐cy7‐anti‐PD‐1 were also added to the two tubes at the same time. All antibodies were purchased from Becton, Dickinson and Company, shaken evenly and incubated without light for 15 min, and then, 400 µl of haemolysin was added. The tubes were immediately shaken evenly and incubated without light for 10 min. Then, 3 ml of saline was added, and the tubes were centrifuged at 1200 rpm for 4 min. The supernatant was discarded, and then, 340 µl of saline was added for the detection by the computer.

FlowJoV10 analysed all the flow cytometry results for phenotypic analysis, and the Bregs were found to be CD19^+^CD24^+^CD38^+^ B cells. For each sample, at least 2 × 10^5^ living cells were gated for calculation.

### 2.3 Statistical analysis

Quantitative data are expressed as the mean plus or minus the standard deviation. The related data for bone marrow and peripheral blood before and after HDs and AML chemotherapy were assessed by ordinary one‐way ANOVA, and the correlation between Bregs and complete remission (CR) of chemotherapy was analysed by Chi‐square (and Fisher's exact) test. Overall survival (OS) and progression‐free survival (PFS) were examined by the Kaplan–Meier method. The follow‐up time was from the beginning of the first collection of bone marrow samples to the last follow‐up time or the end of death of any cause. A *p* < 0.05 was considered significant. All the data were analysed by GraphPad PRISM 8.0 software.

## 3 RESULTS

The demographic characteristics of AML and HDs included in the study are shown in Table 1.

**TABLE 1:** Statistical table of basic characteristics of the patients and healthy donors

Characteristic	HD		AML(*n* = 21)	*p*‐value
	BM (*n* = 15)	PB(n=10)		
Age (mean ± *SD*)	43.27 ± 4.91	46 ± 6.182	51.76 ± 15.92	0.319
Gender				0.814
Male	9	5	13	
Female	6	5	8	
FAB subtypes				
M1	–	–	4	
M2	–	–	9	
M4	–	–	2	
M5	–	–	5	
M7	–	–	1	

Abbreviations: AML, acute myeloid leukemia; BM, bone marrow; HD, healthy donor; PB, peripheral blood.

### 3.1 The expression of PD‐L1 in CD19^+^B cells

**FIGURE 1:**
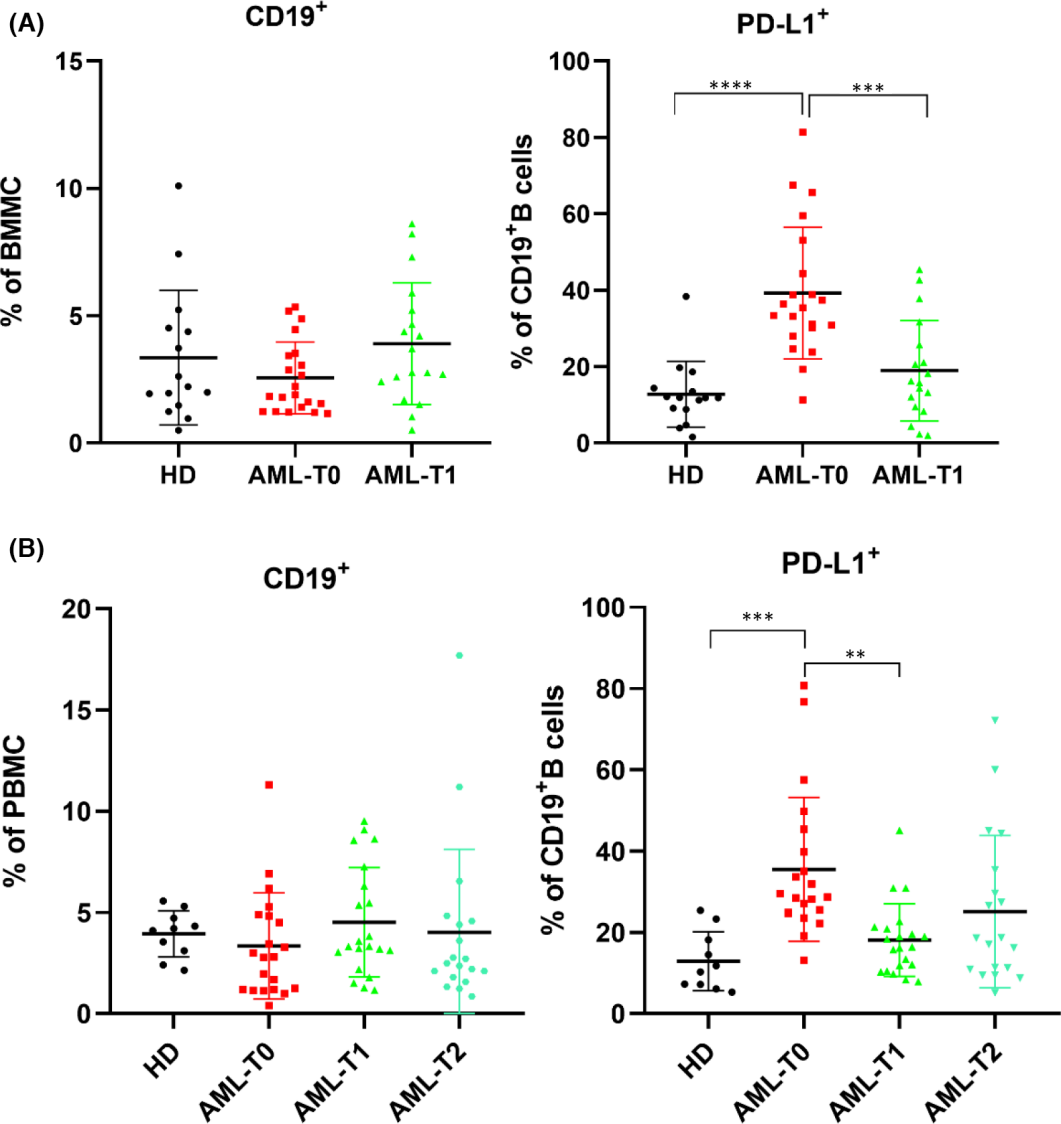
Detection of the expression of CD19 and PD‐L1 by flow cytometry. (A) The percentage of CD19^+^ and its PD‐L1 expression in bone marrow; (B) The percentage of CD19^+^ and its PD‐L1 expression in peripheral blood; HD: healthy donor; AML‐T0: before induction; AML‐T1: 7 days after induction chemotherapy; AML‐T2: 14 days after induction chemotherapy; **p < 0.01; ***p < 0.001; ****p < 0.0001

Our experimental results indicated that there was no significant difference in the number of CD19^+^ B cells between AML patients and HDs in bone marrow or peripheral blood but that the CD19^+^ B cells from AML patients expressed more PD‐L1 than HDs (BM, *p* < 0.001; PB, *p* = 0.001) before treatment. After induction chemotherapy, the difference in PD‐L1 expression on CD19^+^ B cells between patients and HDs vanished, but the difference in PD‐L1 expression on CD19+ B cells of patients before and after chemotherapy was statistically significant (BM, *p* < 0.001; PM, *p* = 0.002), as shown in Figure 1.

### 3.2 The expression of PD‐L1 in Bregs

**FIGURE 2:**
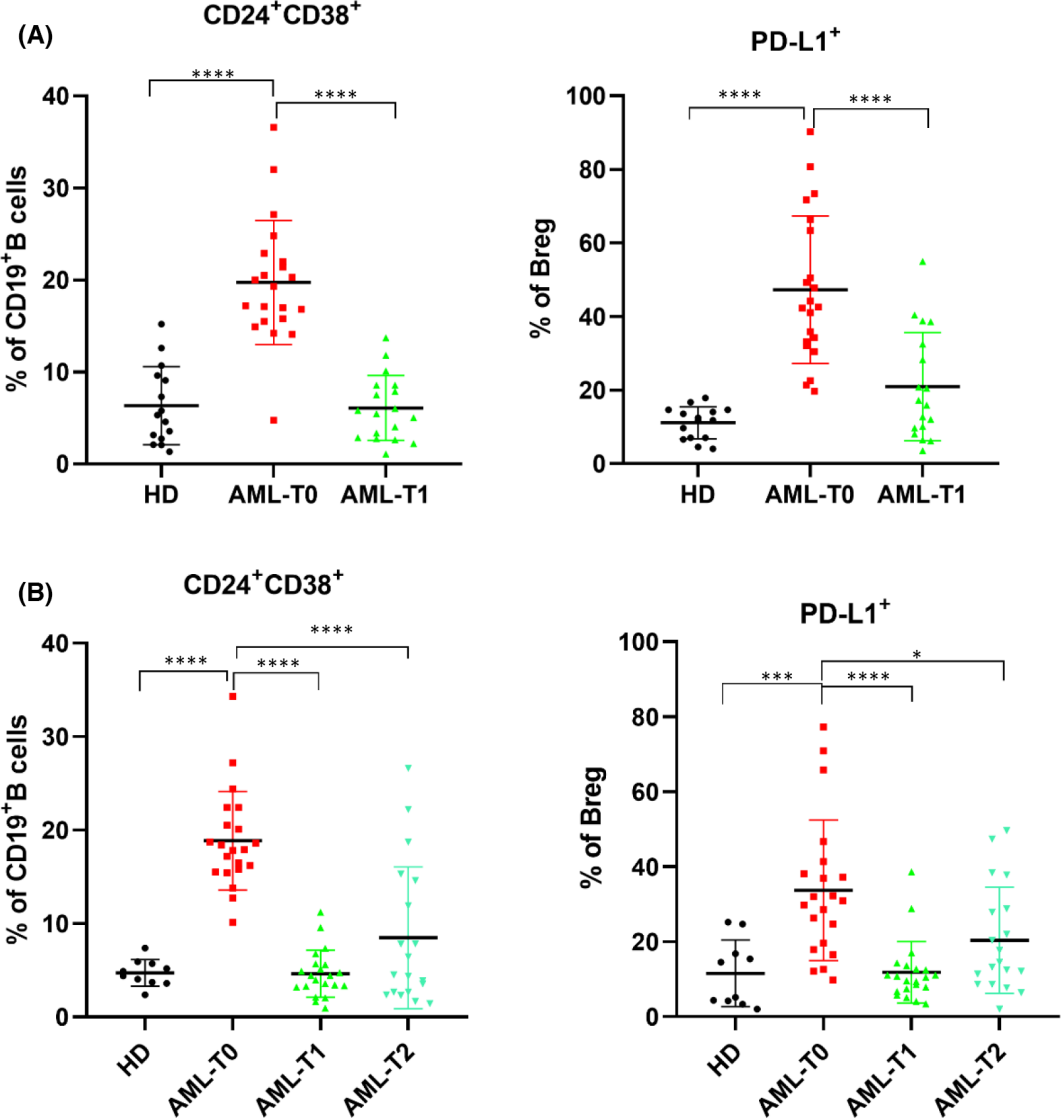
Expression of Bregs and its PD‐L1 in different specimens. (A)The percentage of regulatory B cells in CD19^+^B cells and its PD‐L1 expression in bone marrow; (B) The percentage of regulatory B cells in CD19^+^ B cells and its PD‐L1 expression in peripheral blood; HD: healthy donor; AML‐T0: before induction; AML‐T1: 7 days after induction chemotherapy; AML‐T2: 14 days after induction chemotherapy; *p < 0.05; ***p < 0.001;****p < 0.0001

The proportion of Bregs in the bone marrow and peripheral blood of newly diagnosed AML patients was higher than that of HDs (BM and PB, both *p* < 0.001). The expression of PD‐L1 in Bregs from newly diagnosed AML patients was significantly higher than that in Bregs from HDs (BM and PB, both *p* < 0.001). After receiving induction chemotherapy, PD‐L1 expression was reduced, and there was no significant difference in PD‐L1 expression in Bregs from patients with AML or HDs. For patients, the percentage of PD‐L1 expression on Bregs was significantly different before and after chemotherapy (BM and PB, AML‐T0 *vs*. AML‐T1, both *p* < 0.001; PB, AML‐T0 *vs* AML‐T2, *p* = 0.017), as shown in Figure 2.

### 3.3 The expression of PD‐1 in CD3^+^CD4^+^T cells

**FIGURE 3:**
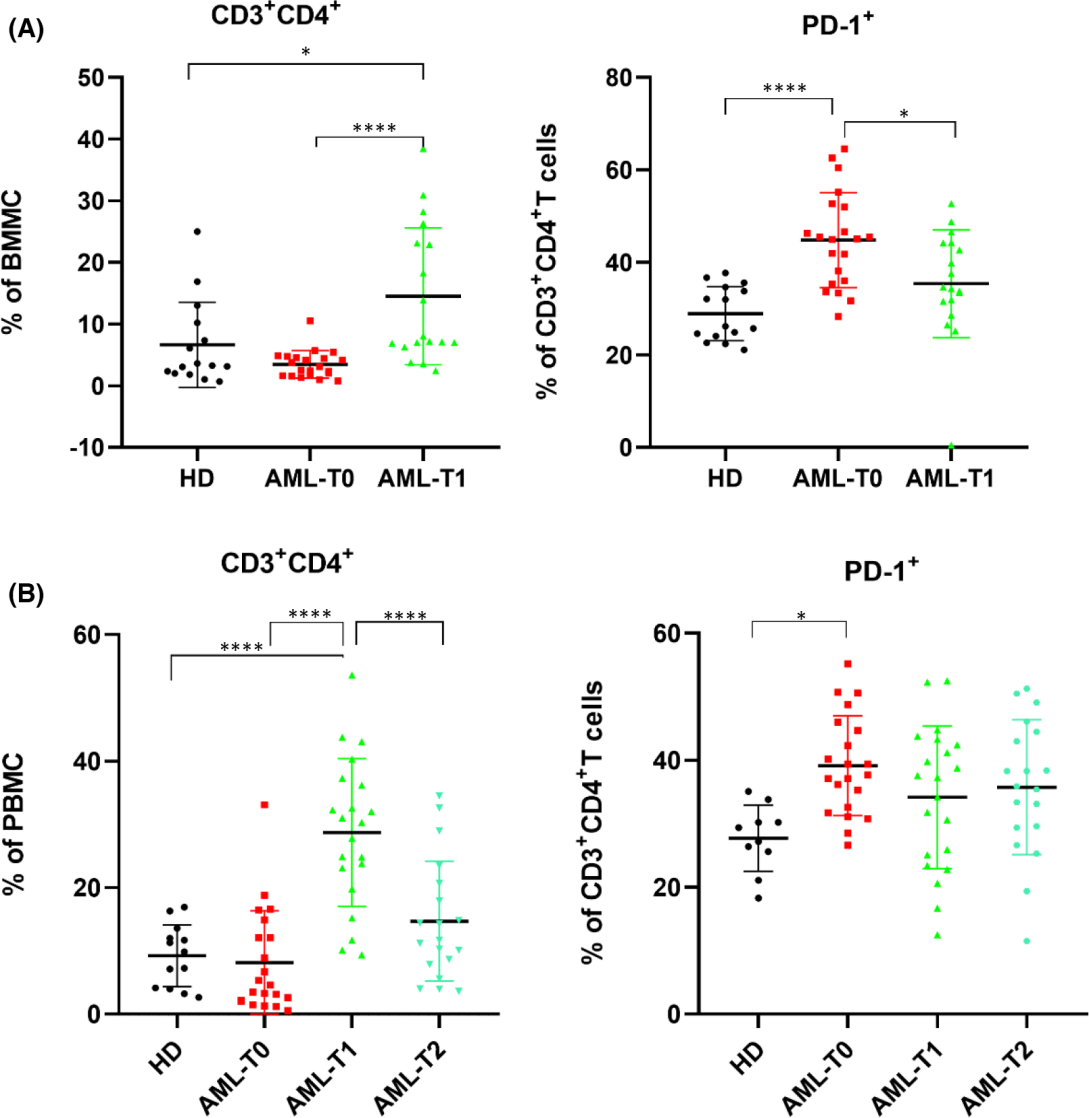
Expression of CD3^+^CD4^+^ T cells and its PD‐1 in different specimens. (A)The percentage of CD3^+^CD4^+^T cells and its PD‐1 expression in bone marrow; (B) The percentage of CD3^+^CD4^+^T cells and its PD‐1 expression in peripheral blood; HD: healthy donor; AML‐T0: before induction; AML‐T1: 7 days after induction chemotherapy; AML‐T2: 14 days after induction chemotherapy; *p < 0.05; ****p < 0.0001

There was no significant difference in the percentage of CD3^+^CD4^+^ T cells between AML patients before chemotherapy and HDs in bone marrow(*p* = 0.429) or peripheral blood (*p* = 0.987). After chemotherapy, the fraction of CD3^+^CD4^+^ T cells was much higher than that before chemotherapy (BM and PB, both *p* < 0.001), and there were significant differences between AML patients and HDs (BM, *p* = 0.011; PB, HDs *vs*. AML‐T1, *p* < 0.001). In addition, we discovered that the fraction of CD3^+^CD4^+^ T cells in peripheral blood was lower at 14 days after chemotherapy than at 7 days after chemotherapy, and the difference was statistically significant (*p* < 0.001). It was still higher than that before chemotherapy and HDs, but the difference was not statistically significant (AML‐T0 *vs*. AML‐T2, *p* = 0.124; HDs *vs*. AML‐T2, *p* = 0.366). As demonstrated in Figure 3, PD‐1 expression was much higher in CD3^+^CD4^+^ T cells from bone marrow and the peripheral blood of newly diagnosed AML patients than in HDs (BM, *p* < 0.001; PB, *p* = 0.014). In bone marrow, the percentage of PD‐1 expression in CD3^+^CD4^+^ T cells before chemotherapy was higher than that after chemotherapy (*p* = 0.012), but the difference was not significant in peripheral blood (*p* = 0.332).

The expression of all indexes in the peripheral blood and bone marrow samples is shown in Tables 2 and 3.

**TABLE 2:** Summary of the expression of various indexes in bone marrow

	HD	AML‐T0	AML‐T1
CD19^+^	3.351±2.641	2.559±1.41	3.897±2.392
CD19^+^PD‐L1^+^	12.77±8.642	39.24±17.28	18.96±13.14
CD19^+^CD24^+^CD38^+^	6.337±4.249	19.73±6.734	6.074±3.526
CD19^+^CD24^+^CD38^+^PD‐L1^+^	11.13±4.375	47.3±20.02	20.94±14.72
CD3^+^CD4^+^	6.636±6.927	3.462±2.218	14.52±11.11
CD3^+^CD4^+^PD−1^+^	28.95±5.846	44.85±10.27	35.41±11.69

Abbreviations: AML‐T0, T0: before induction; AML‐T1,7 days after induction chemotherapy; HD, healthy donor.

**TABLE 3:** Summary of the expression of various indexes in peripheral blood

	HD	AML‐T0	AML‐T1	AML‐T2
CD19^+^	3.943±1.144	3.341±2.614	4.512±2.712	4.021±4.091
CD19+PD‐L1^+^	12.94±7.236	35.51±17.67	18.12±8.93	25.14±18.71
CD19+CD24+CD38^+^	4.698±1.419	18.85±5.285	4.614±2.514	8.457±7.602
CD19^+^CD24^+^CD38^+^PD‐L1^+^	11.56±8.923	33.68±18.72	11.84±8.197	20.37±14.17
CD3^+^CD4^+^	9.232±4.896	8.133±8.214	28.72±11.67	14.67±9.489
CD3^+^CD4^+^PD−1^+^	27.73±5.224	39.14±7.88	34.16±11.22	35.74±10.62

Abbreviations: AML‐T0, before induction; AML‐T1, 7 days after induction chemotherapy; AML‐T2, 14 days after induction chemotherapy; HD, healthy donor.

### 3.4 Patients with high PD‐L1 expression have a poor prognosis

**FIGURE 4:**
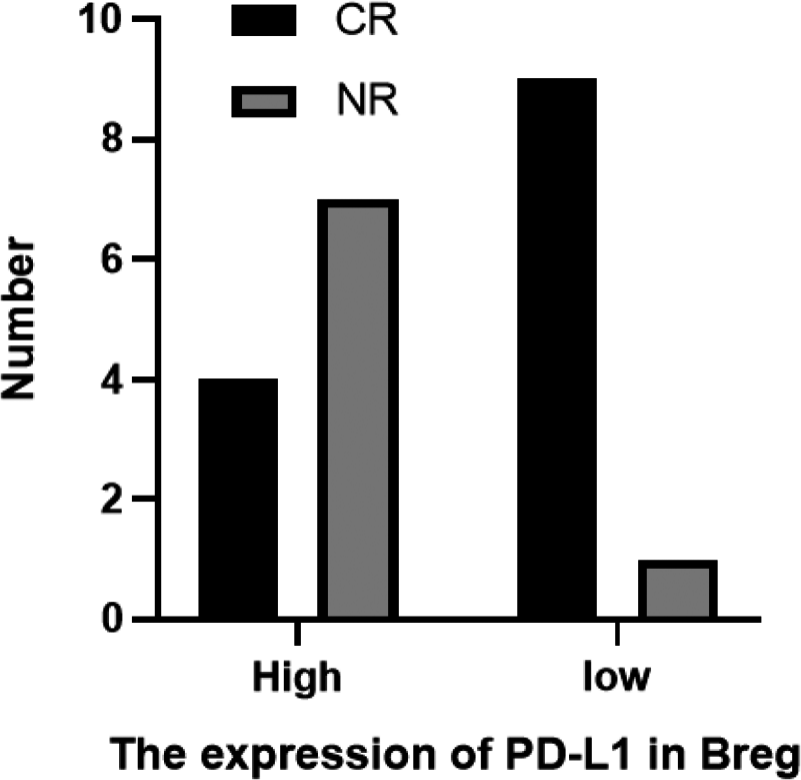
Effect of PD‐L1 expression in bone marrow on prognosis

**FIGURE 5:**
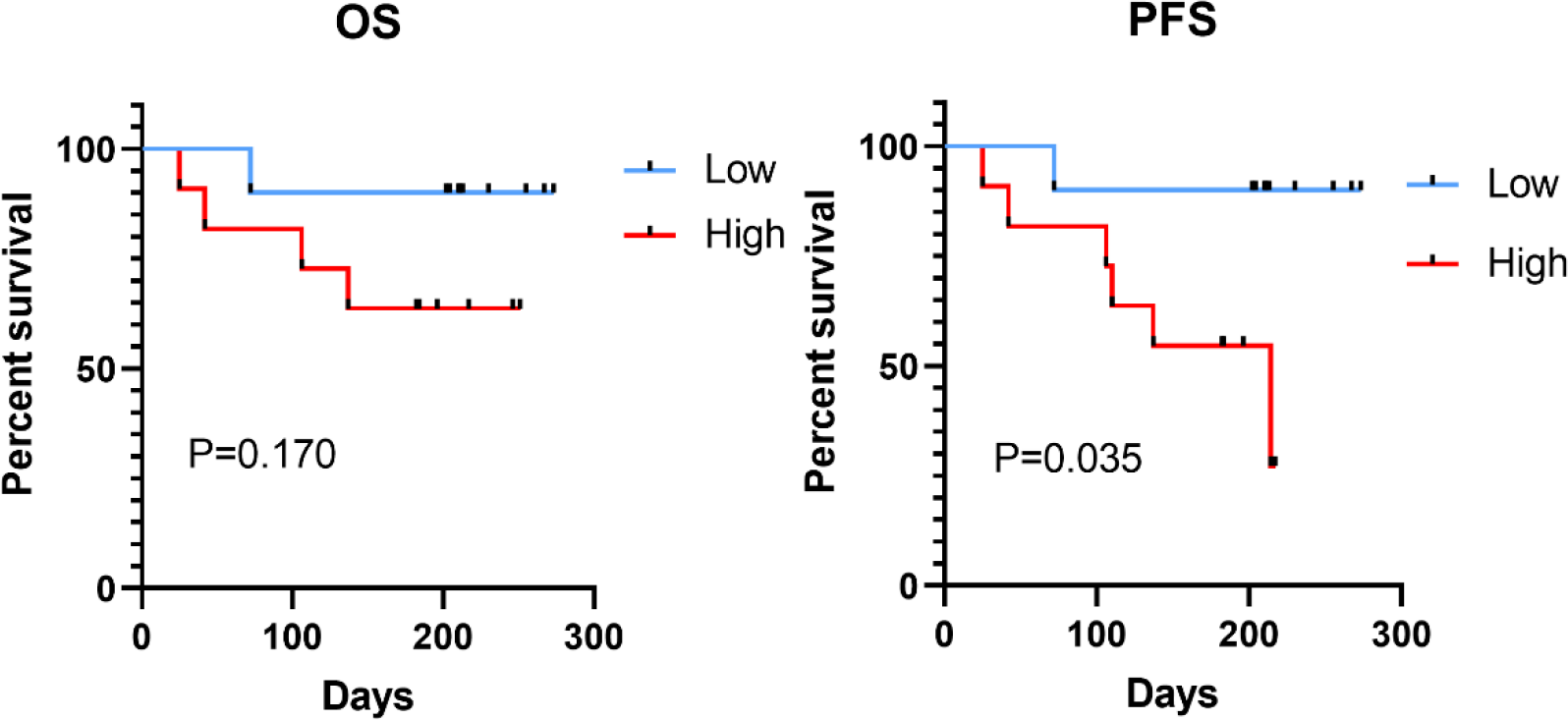
Overall survival and progression‐free survival curves of the PD‐L1 high expression group and the PD‐L1 low expression group in AML

To determine whether a high PD‐L1 expression is associated with a poor prognosis, we split the patients into high and low expression groups based on the median PD‐L1 expression in Bregs. The basic statistics of the two groups are shown in Table 4. The remission rates of the two groups following induction treatment were compared. Patients with a high PD‐L1 expression had a poor prognosis, and the difference between the two groups was statistically significant(*p* = 0.024), as shown in Figure 4. After follow‐up, there was no significant difference in OS between the two groups (*p* = 0.170), but PFS decreased significantly in the high expression group (*p* = 0.035) (Figure 5).

**TABLE 4:** Statistical table of the basic characteristics of the PD‐L1 high expression group and the low expression group in AML

Characteristic	Low (*n* = 10)	High (*n* = 11)	*p*‐value
Age (mean ± *SD*)	52.1 ± 15.18	46.55 ± 16.77	0.430
Gender			0.669
male	5	7	
Female	5	4	
WBC count	20.42 ± 30.87	68.31 ± 89.85	0.126
FAB subtypes			0.112
M1	2	2	
M2	4	5	
M4	1	1	
M5	2	3	
M7	1	‐	
Risk stratification			0.384
Low risk	4	3	
Medium risk	5	3	
High risk	1	5	
Chemotherapy regimen			0.569
IA	9	8	
Dexitabine + HAG	1	1	
DA	–	1	
others	–	1	
Therapeutic effect			0.024
CR	9	4	
NR	1	7	

## 4 DISCUSSION

Cancer progression is a multistep process involving tumour activity and the immune system. The immune system is compromised in the majority of cancer patients.^17^ Regulatory B cells play an essential role in the immune system. Our previous study showed that Bregs were highly expressed in patients with AML and related to poor prognosis.^9^ Furthermore, we found that Bregs can participate in the immune regulation of T cells, while it did not inhibit T cells via cytokines, including IL‐10 (Data unpublished). In this study, we found that PD‐L1 expression in Bregs were significantly higher in AML patients than that in healthy donors, and both the number of Bregs and PD‐L1 expression decreased significantly after chemotherapy. Chang et al. confirmed that myelogenous suppressor cells (MDSCs) can promote the transformation of immature B cells into Bregs in the tumour microenvironment, and in this process, MDSCs can mediate the transfer of membrane‐bound PD‐L1 to Bregs in the form of secretory vesicles, thus increasing the expression of PD‐L1 in Bregs and mediating immunity through the PD‐L1 pathway to inhibit T‐cell function.^18^ Therefore, in addition to IL‐10, the immunosuppressive function mediated by PD‐L1 in Bregs has attracted attention in recent years. Khan et al. proved that PD‐L1 could inhibit the proliferation of PD‐1^+^ Tfh cells and downregulate the humoral immune response in a mouse model and in vitro cell experiments.^19^ Zhang et al. confirmed that tumour‐infiltrating B cells express more PD‐L1 than splenic B cells in a mouse model. These B cells can suppress the growth of CD4^+^ and CD8^+^ T cells, and anti‐PD‐L1 antibodies can restore this inhibitory effect.^20^ In addition, a number of studies have demonstrated that cancer immunotherapy strategies that block the interaction between PD‐1 and PD‐L1 can restore T‐cell activity and lead to tumour regression in both humans and animals.^17^, ^21^, ^22^, ^23^ Although the immune mechanism of AML is related to T cells, our experimental results indicate that there is no significant difference in the number of T cells in bone marrow or the peripheral blood between healthy donors and AML patients, but there is a significant increase in the expression of PD‐1 on T cells in AML patients, implying that T cells have an abnormal function in AML patients that may be related to PD‐1. Therefore, we speculated that PD‐L1^+^ Bregs support immune escape by inhibiting the function of normal T cells through PD‐L1, leading to AML progression. However, further confirmation is needed both in vitro and in vivo. These findings related to PD‐L1 and PD‐1 represent a highly promising breakthrough that could aid in developing new approaches for cancer treatment. With a more in‐depth understanding of the role of immune checkpoints in inhibiting T‐cell activation, the role of immune checkpoint inhibitors in the treatment of cancer is also becoming clearer.^10^ Our experiment also laid a foundation for the application of immune checkpoint inhibitors in patients with AML.

Our study still has some limitations, which should be noted; at this stage, our experiment is limited to describing experimental phenomena, but cell experiments and animal experiments are being planned as a next step, and the relevant experimental results will be reported in the next few years. Our experimental clinical data remain insufficient, and the role of PD‐L1 in patient prognosis has not yet been fully elucidated.

## 5 CONCLUSIONS

In summary, our study confirmed the increased expression of PD‐L1 in Bregs in patients with AML, which may be related to immune escape. These experiments lay the foundation for further exploration of the immune escape mechanism of AML and provide insights and evidence for the further study of AML treatment with immune checkpoint inhibitors.

## AUTHOR CONTRIBUTION

**Yingqing Shi:** Data curation (lead); Formal analysis (lead); Investigation (lead); Writing – original draft (lead); Writing – review & editing (lead). **Zhuogang Liu:** Project administration (lead); Supervision (lead); Writing – original draft (supporting); Writing – review & editing (supporting). **Hongtao Wang:** Funding acquisition (lead); Methodology (lead); Resources (lead); Writing – original draft (supporting); Writing – review & editing (supporting).

## CONFLICT OF INTEREST

The authors declare no conflict of interest.

## Data Availability

The data that support the findings of this study are available on request from the corresponding author. The data are not publicly available due to privacy or ethical restrictions.
